# Differential DNA Methylation in Relation to Age and Health Risks of Obesity

**DOI:** 10.3390/ijms160816816

**Published:** 2015-07-24

**Authors:** María Luisa Mansego, Fermín I. Milagro, María Ángeles Zulet, María J. Moreno-Aliaga, José Alfredo Martínez

**Affiliations:** 1 CIBERobn, Fisiopatología de la Obesidad y la Nutrición, Institute of Health Carlos III, Madrid, Spain and Department of Nutrition, Food Science and Physiology, Nutrition Research Center, University of Navarra, Pamplona 31008, Spain; E-Mails: mlmansego@unav.es (M.L.M.); fmilagro@unav.es (F.I.M.); mazulet@unav.es (M.A.Z.); mjmoreno@unav.es (M.J.M.-A.); 2 Navarra Institute for Health Research (IdiSNA), Pamplona 31008, Spain

**Keywords:** epigenetics, BMI, gene expression, *GPR133*, *ITGB5*, *PRKCZ*, *PI4KB*, *ELOVL2*

## Abstract

The aim of this study was to evaluate whether genome-wide levels of DNA methylation are associated with age and the health risks of obesity (HRO); defined according to BMI categories as “Low HRO” (overweight and class 1 obesity) *versus* “High HRO” (class 2 and class 3 obesity). Anthropometric measurements were assessed in a subsample of 48 volunteers from the Metabolic Syndrome Reduction in Navarra (RESMENA) study and 24 women from another independent study, Effects of Lipoic Acid and Eicosapentaenoic Acid in Human Obesity (OBEPALIP study). In the pooled population; the methylation levels of 55 CpG sites were significantly associated with age after Benjamini-Hochberg correction. In addition, DNA methylation of three CpG sites located in *ELOVL2*; *HOXC4* and *PI4KB* were further negatively associated with their mRNA levels. Although no differentially methylated CpG sites were identified in relation to HRO after multiple testing correction; several nominally significant CpG sites were identified in genes related to insulin signaling; energy and lipid metabolism. Moreover, statistically significant associations between BMI or mRNA levels and two HRO-related CpG sites located in *GPR133* and *ITGB5* are reported. As a conclusion, these findings from two Spanish cohorts add knowledge about the important role of DNA methylation in the age-related regulation of gene expression. In addition; a relevant influence of age on DNA methylation in white blood cells was found, as well as, on a trend level, novel associations between DNA methylation and obesity.

## 1. Introduction

Obesity plays a crucial role in metabolic syndrome features onset, and it has been defined as a chronic disease associated with important cardiorespiratory and cardiometabolic risks [1]. The predisposition to gain excessive weight and develop obesity as a result of the intake of energy-rich diets is partly controlled by specific genes [2]. In some studies, between 40% and 70% of the body weight variability has been attributed to genetic inheritance, being the number of associated genes continuously increasing due to Genome-Wide Association Studies (GWAS) [3]. The increase in obesity prevalence may also be the result of epigenetic changes [4,5]. Epigenetics studies the inheritable and reversible phenomena that affect gene expression without altering the underlying base pair sequence [6]. Thus, the same nucleotide sequence in two individuals can be differently expressed or not depending on specific epigenetic marks [7].

DNA methylation occurs preferentially in sequences whose pattern is a cytosine followed by a guanine residue, called CpG dinucleotides. These CpG sites are especially common in the promoter regions of many genes and, when methylated, tend to be associated with gene silencing [8]. These epigenetic marks are not permanent over time and many factors, such as nutrition, inflammation, physical activities, oxidative stress, hypoxia, smoking, sex or age, induce changes in the epigenome, contributing to its plasticity throughout life [9,10].

Several studies have also shown that changes in the epigenome vary during the different life cycle stages [11,12]. Thus, it has been reported that epigenetic marks at the early age are responsible for controlling gene expression during adulthood [13]. In this sense, different epigenetic patterns associated with aging seem to occur, which are related to changes in regulatory gene expression and metabolic functions. Aging could lead to genome-wide demethylation in certain gene coding regions [14], although, more often, aging has been related to increased methylation of certain CpGs in specific gene families, CpG islands [15] and bivalent chromatin domains [16]. These epigenetic modifications could be implicated in the onset of cancer and other diseases associated with advancing age, and also appear to depend on the gene and tissue analyzed. For example, studies in liver and visceral adipose tissue have revealed differences in DNA methylation with age at >5% of sites analyzed (from a total of one thousand randomly selected loci), many of them near genes that are involved in metabolic regulation, suggesting a potential role in the pathogenesis of age-related diseases [17].

Moreover, obesity is not only an aesthetic problem due to inadequate dietary and sedentary lifestyle habits, since excessive fat accumulation greatly raises the risk for other health problems, such as coronary heart disease, type 2 diabetes, hypertension, metabolic syndrome or reproductive problems [18]. All these complications are included under the name of health risk of obesity (HRO). The National Institutes of Health guidelines indicate that the HRO increases in a graded fashion when moving from the normal-weight through obese BMI categories [19]. In addition to a predisposing genetic makeup to easier weight gain and fat deposition, a lot of recognized scientific evidence has theorized about the roles of other putative determinants. Several investigations aiming to understand energy metabolism have been performed considering the potential involvement of epigenetics and perinatal programming [13,20,21]. Indeed, inheritance-oriented investigations concerning gene-nutrient interactions on energy homeostasis processes and metabolic cell functions are extending to all clinically chronic relevant diseases, such as type 2 diabetes, cardiovascular events, obesity and associated features of metabolic syndrome.

The aim of this study was to evaluate whether genome-wide levels of DNA methylation in white blood cells are associated with age and the HRO, defined as the risk of obesity-related health problems, in two unrelated Spanish cohorts.

## 2. Results and Discussion

We have conducted a pilot study to examine the associations between DNA methylation and age, as well as HRO, in order to better understand epigenetic contributions to health in two Spanish populations, where these associations, to our knowledge, have not yet been examined.

A total of 73 participants, 35.6% men, were suitable for the analysis. The general features of subjects grouped by study are shown in Table 1. As expected, the “High HRO” group evidenced greater levels (*p* < 0.05) of body weight, BMI and waist circumference than the “Low HRO” group (Table 1). Age and anthropometric measurements were significantly lower in the “Effects of Lipoic Acid and Eicosapentaenoic Acid (EPA) in Human Obesity” (OBEPALIP) population compared with the Metabolic Syndrome Reduction in Navarra (RESMENA) study (*p*-value < 0.001). These two cohorts differed in the inclusion criteria. The OBEPALIP study recruited only women without metabolic syndrome from 20 to 45 years old, while the RESMENA study had broader criteria (both genders aged between 35 and 70 years old with a high prevalence of metabolic syndrome). Moreover, anthropometric differences between the two populations were mainly due to age, the presence of metabolic syndrome and gender. Women from OBEPALIP study could be considered more metabolically healthy overweight and obese subjects (without other complications), while most subjects from RESMENA population had metabolic syndrome (81%). Metabolically healthy obese individuals display less visceral adipose tissue, smaller adipocytes, and a reduced inflammatory profile when compared with metabolically unhealthy obese individuals [22]. Healthy obese subjects are also at a lower risk thereof than individuals who are both unhealthy and obese [23]. The relatively low risk of cardiovascular disease among metabolically healthy people with respect to unhealthy obese people has been attributed to differences in white adipose tissue function between both groups of patients [24].

**Table 1:** General characteristics of the studied subjects.

Variables	Pooled Population			RESMENA Study			OBEPALIP Study		
	TP	LR	HR	TP	LR	HR	TP	LR	HR
***n***	73	40	33	48	18	30	25	22	3
**Age (year)**	45 (10)	41 (10)	49 (10) ^+^	48 (10)	47 (10)	49 (10)	37 (7) *	37 (8)	42 (2)
**Sex (M/F)**	26/47	9/31	17/16 ^+^	26/22	9/9	17/13	0/25	0/22	0/3
**Weight (Kg)**	95.5 (17.8)	85.8 (10.4)	107.0 (18.0) ^+^	102.4 (17.9)	90.5 (11.6)	109.1 (17.4) ^+^	82.7 (7.6) *	82.3 (7.9)	86.0 (4.0)
**BMI (kg/m^2^)**	34.6 (4.1)	31.5 (2.3)	38.1 (2.7) ^+^	36.2 (3.8)	32.4 (2.7)	38.2 (3.8) ^+^	31.5 (2.7) *	30.9 (2.3)	36.0 (0.6) ^+^
**WC (cm)**	106.0 (13.4)	98.1 (8.8)	115.3 (11.9) ^+^	112.0 (12.5)	102.5 (10.2)	117.3 (10.5) ^+^	94.7 (5.7) *	94.6 (5.8)	95.3 (5.7)
**Metabolic Syndrome ^1^**	39	12	27	39	12	27	0 *	0	0
**Smoking**	12	7	5	8	3	5	4	4	0

Data are expressed as mean (standard deviation). Abbreviations: TP, total population; LR, Low HRO; HR, High HRO; *n*, number of subjects; BMI, body mass index; WC, waist circumference; ^1^ The metabolic syndrome was diagnosed following the ATP III criteria; * Significant differences between the two populations, *p*-value < 0.001; ^+^ Significant differences between low and high HRO, *p*-value < 0.05.

### 2.1. Identification of CpG Sites Differentially Methylated with Age in WBC

**Figure 1:**
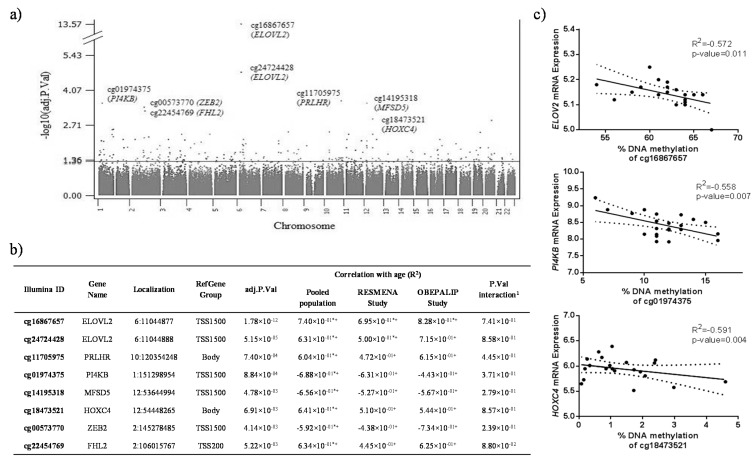
Associations between age and DNA methylation in different CpGs. (**a**) Manhattan plot of genome-wide *p*-values from the pooled population; (**b**) Adjusted *p*-values and Spearman correlation coefficients of the selected CpG sites with respect to age. CpGs are ranked by adjusted *p*-value, from smallest to largest. The number of subjects included in the pooled population, RESMENA study and OBEPALIP, were 73, 48 and 25, respectively. ^1^ *p*-value of age-cohort interaction analyzed with a linear regression. * denotes statistical significance at adjusted *p*-value < 0.05; ^+^ denotes statistical significance at raw *p*-value < 0.01; (**c**) Graphical representation of the correlation between DNA methylation levels of the selected CpG sites and mRNA expression of the respective genes in RESMENA study (*n* = 24). Data are presented as linear regression (solid straight line) graph and 95% confidence interval (dotted lines). Localization: Chromosome on which the target locus is located and genomic position of C in the CpG dinucleotide; RefGene Group: Gene region feature category; adj.P.Val: adjusted *p*-value for multiple testing.

In this study, the white blood cell (WBC) DNA methylation profile was analyzed in 73 subjects using Illumina Infinium HumanMethylation450 BeadChip (Illumina, San Diego, CA, USA). The age range was 21–58 years old. Linear regression analysis identified 54 CpG sites associated with age (Table S1). The top 8 significant loci located within or nearby to the CpG islands of *ELOVL2*, *PRLHR*, *PI4KB*, *MFSD5*, *HOXC4*, *ZEB2* and *FHL2* genes had the smallest *p*-value below the Benjamini-Hochberg threshold (≤0.05) adjusted for gender, smoking, metabolic syndrome, the research group that made each study, T cell (CD8+), T cell (CD4+), B cells and random batch effect (Figure 1a). The same CpGs showed the highest Spearman’s coefficients (Figure 1b) between DNA methylation levels and age in the WBC from the pooled population and in the two independent populations (RESMENA and OBEPALIP studies). The patterns of results in both cohorts were comparable (the correlation coefficients describe the same direction (positive or negative) and statistical significance at raw *p*-value <0.01). Moreover, the possible interaction effects between cohorts and age on DNA methylation levels that represent the combined effects of factors on the dependent measure were tested. No interaction effects were found. Thus, the impact of age and methylation is independent of the cohorts.

DNA methylation is well known to change during aging [25]. Recent studies have demonstrated the presence of age-related CpG sites, which are characterized by a global loss of DNA methylation during aging [26]. However, some genes become hypermethylated with age [26]. The methylation levels of the CpG sites of *ELOVL2*, *PRLHR*, *HOXC4*, and *FHL2* positively correlated with age. In this sense, DNA methylation levels could be used to estimate age [27]. Biological clocks, such as the epigenetic clock, are promising biomarkers of aging [28]. However, this pilot research requires further studies to replicate the age-related associations and find the predictive value of the estimate age.

Increased DNA methylation of certain genomic regions may be involved in the silencing of gene transcription [29]. Therefore, it was tested if these selected CpG sites with increased DNA methylation were associated with decreased mRNA expression of genes located near the CpG sites in the RESMENA cohort. The DNA methylation levels of three of the selected CpG sites (cg16867657, cg01974375 and cg18473521) showed a statistically significant negative correlation with the mRNA levels of the respective genes (*ELOV2*, *PI4KB* and *HOXC4*) in the same cells (WBC) of the screened subjects (Figure 1c).

The methylation levels of CpG sites located in genes like *ELOVL2* and *FHL2*, have been strongly correlated with age in previous genome-wide methylation studies [30,31]. *ELOVL2* encodes a transmembrane protein involved in the synthesis of long ω3- and ω6-polyunsaturated fatty acids (PUFA) [32]. Considering that PUFAs are involved in crucial biological functions including energy production, inflammation, and maintenance of cell membrane integrity, it is possible that *ELOVL2* methylation plays a role in the aging process through the regulation of different biological pathways.

By using a linear mixed model in a transcriptomic approach, the gene expression of *MFSD5* in skin was associated with age [33]. *MFSD5* (major facilitator superfamily domain containing 5) encodes a protein that facilitates the transport across cytoplasmic or internal membranes of molybdate anion. However, the age-related mechanism that affects the expression of this gene remains to be elucidated.

A CpG site of *HOXC4* was significantly associated with age. *HOXC4* is a member of the homeobox (HOX) family of master transcription factors crucial in morphogenesis and development [34]. The expression of many HOX genes, including *HOXC4*, declines with age, even prior to adulthood [35]. An association of HOX genes with longevity has been proposed [36], although there is not direct evidence so far linking *HOXC4* to human aging. *ZEB2* is another age-associated gene. This gene (also known as *SIP1*) is a member of the Zfh1 family of 2-handed zinc finger/homeodomain proteins. Its relevance to tumor progression has been studied in several forms of human cancer [37], although the association of *ZEB2* with age has not been previously reported. Lastly, phosphatidylinositol 4-kinase beta (PI4KB) is a soluble enzyme shuttling between the cytoplasm and the nucleus, which regulates the trafficking from the Golgi system to the plasma membrane [38]. Although this gene has been recently described as a candidate age-associated genomic region [39], its involvement in aging is still unclear.

The Gene Ontology (GO) analysis of these CpG sites differentially methylated with age showed processes related to apoptosis, signal transduction and transcriptional regulation, rhythmic process, DNA repair and chromatin remodeling, cell differentiation or metabolic processes. However, no significant terms were found in GO enrichment analysis.

### 2.2. Identification of CpG Sites Differentially Methylated in Relation to the Health Risks of Obesity (HRO)

**Figure 2:**
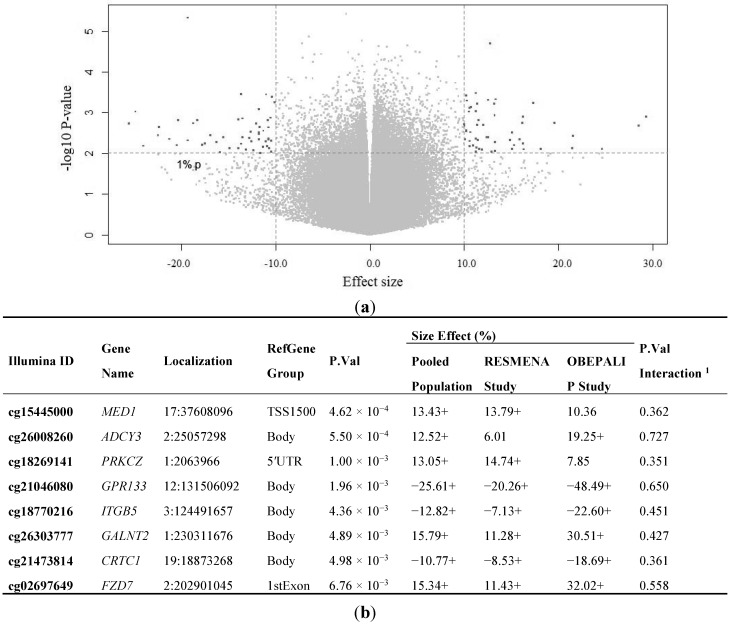
Identification of CpGs and genes differentially methylated between “Low HRO” and “High HRO” subjects. (**a**) Volcano plot of the 358,639 CpG sites of raw *p*-values log *versus* mean methylation differences (effect size) comparing Low HRO group to High HRO groups. Hypermethylation means more methylation in the Low HRO group compared to the High HRO group, and hypomethylation refers to less methylation in the Low HRO group than in the High HRO group. CpG sites hypermethylated (*n* = 44) and hypomethylated (*n* = 41) are shown in black; CpG sites that were not differentially methylated (raw *p*-value > 0.01) are shown in grey; (**b**) *p*-values and effect size of the selected CpG sites between groups. CpGs are ranked by *p*-value, from smallest to largest. ^1^ *p*-value of HRO-cohort interaction analyzed with ANOVA. +, denotes statistical significance at raw *p*-value < 0.01.

We identified 85 CpG sites (Figure 2a) differentially methylated (mean absolute methylation difference ≥ 10%; raw *p*-value < 0.01) between “Low HRO” (overweight and class 1 obesity) and “High HRO” (class 2 and 3 obesity). However, none of these CpGs remained statistically significant after Benjamini-Hochberg correction. Forty-one CpG sites were hypomethylated and 44 hypermethylated in the “Low HRO” group compared to the “High HRO” group (Table S2), as shown in the Volcano plot (Figure 2a). The GO analysis revealed that the genomic regions where these differentially methylated CpG sites are located have been involved in different processes related to apoptosis, transcriptional regulation and inflammatory response, cell cycle, oxidative stress response, energy reserve metabolic processes, insulin receptor signaling pathway and carbohydrate and lipid metabolic processes (Table S2).

Eight CpG sites (Figure 2b) were selected for further demonstrating whether the changes in DNA methylation were accompanied by changes in gene expression. The selection of these CpG sites was based on two conditions: the methylation differences between “Low HRO” and “High HRO” were ≥10% and *p*-value ≤0.01, and previous reports associating these genes with obesity [40,41,42,43,44,45,46,47]. The patterns of results in both cohorts were comparable in most of the selected CpG sites (the same size effect direction (positive or negative) and statistical significance at raw *p*-value <0.01). The possible interaction effects between cohorts and HRO on DNA methylation levels were also tested. No interaction effects were found. Thus, the impact of HRO and methylation is independent of the cohorts.

**Figure 3:**
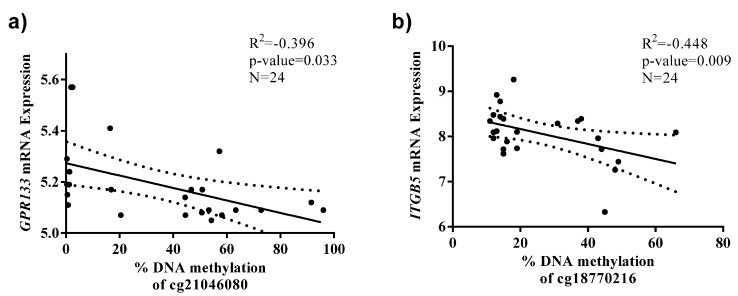
Associations between CpG sites that exhibit differential DNA methylation between the HRO groups and the expression levels of their respective genes in WBC from the RESMENA cohort. Data are presented as linear regression (solid straight line) graph and 95% confidence interval (dotted lines). (**a**) cg21046080 and *GPR133*; (**b**) cg18770216 and *ITGB5*.

The expression analysis of these selected genes identified that the DNA methylation levels at CpG sites measured by the probes cg21046080 and cg18770216 were negatively correlated with the expression of *GPR133* and *ITGB5*, respectively, in WBC from the RESMENA cohort (*n* = 24) (Figure 3).

Moreover, a linear regression model was used to analyze the potential associations in relation to each domain of variables (DNA methylation, age, gender, smoking, metabolic syndrome, the research group that made each study and batch effect) and BMI as dependent variable in the pooled population (*n* = 73). Thus, the predictors of the model (metabolic syndrome and DNA methylation levels) explained up to 40% of the variation of the BMI in the case of cg18269141. This CpG is located in *PRKCZ* gene, a gene with a potential role in obesity and/or related traits [41]. The *PRKCZ* methylation in human adipose tissue is modified after gastric bypass and weight loss [41] and the hypermethylation of this gene may be involved in the pathogenesis of type 2 diabetes [48] (Table 2).

**Table 2:** Lineal regression analyses showing the contribution of DNA methylation to the variation of BMI adjusted by age, gender, smoking, metabolic syndrome, the research group that made each study, and batch effect in the pooled population (*n* = 73).

BMI as Dependent	Standardized Beta Coefficient	Unstandardized B Coefficient (95% CI)	*p*-Value ^a^	*R*^2^
***cg15445000_MED1***	***−0.249***	***−6.61 (−11.78; −1.43)***	***0.013***	***0.335***
***cg26008260_ADCY3***	***−0.332***	***−103.66 (−168.75; −38.6)***	***0.002***	***0.349***
***cg18269141_PRKCZ***	***−0.356***	***−8.73 (−13.68; −3.78)***	***0.003***	***0.397***
***cg21046080_GPR133***	***0.278***	***5.11 (1.68; 10.01)***	***0.017***	***0.292***
***cg18770216_ITGB5***	***0.211***	***5.26 (0.21; 10.31)***	***0.041***	***0.299***
**cg26303777_*GALNT2***	0.122	2.61 (−1.91; 7.11)	0.252	0.360
**cg21473814_*CRTC1***	−0.039	−1.13 (−7.28; 5.20)	0.771	0.270
**cg02697649_*FZD7***	−0.103	−2.08 (−6.21; 2.05)	0.318	0.279

Adjusted *R*^2^ and all independent variables included in each model are presented in the table; Bold and italic style show statistically significant *p*-values; ^a^ *p*-value of Beta coefficient for DNA methylation levels; CI: Confidence interval.

DNA methylation is an epigenetic event implicated in several human diseases by altering gene expression [49], these processes being suggested as a potential contributing factor to cancer risk [50]. Recent researches have shown that differential variability in methylation is also an important feature of obesity [51] and, for example, some genes have been revealed to be hypermethylated in lean compared with obese subjects [52]. On the other hand, the methylation of specific genomic regions could be related to the response to a weight loss intervention, both in obese adults [4,53,54] and in adolescents [55]. Concerning the metabolic complications of obesity, it has been recently reported that the leukocyte methylome is altered in obesity associated with metabolic disturbances, including differences in 23 genes known to be associated with obesity, liver fat, type 2 diabetes and metabolic syndrome [56]. Moreover, platelet mitochondrial DNA methylation has been recently related to cardiovascular risk, although not with BMI [57].

In our study, we have described significant correlations between BMI and the methylation levels of five CpGs, which may explain some of the BMI variation observed in our studied population. In addition, we have found negative correlations between the methylation of two CpG sites (cg21046080 and cg18770216) and the mRNA levels of the respective genes (*GPR133* and *ITGB5*) in WBC of the RESMENA study. Existing data are consistent with a role for these genes in body weight regulation. They are specifically expressed in glands that hormonally are involved in the control of body weight and fat (*GPR133* in adrenal glands) [40] or are up-regulated in obese children (*ITGB5*) [42]. Henegar *et al.* [58] also reported that *ITGB5* and other genes encoding the members of the integrin family were significantly induced and co-expressed in adipose tissue from obese adults.

Our study has certain limitations. First, since the nature of this study is cross-sectional, we can only report associations between age/HRO and DNA methylation even if controlling for several potential covariates but not a causal relationship. Another limitation of this study is that, although the sample size is adequate from the standpoint of an initial association discovery, further replications would be needed in independent and larger samples. Furthermore, no blood cell count was carried out, resulting in a possible limitation in the interpretation of DNA methylation levels due to the influence of the tissue heterogeneity in epigenetic studies related to age and obesity [27,59]. Nevertheless, one of the aims of the present work was to use WBC as a source of easily isolated biomarkers of HRO, as other studies have previously reported [54,60]. Moreover, we performed an additional analysis to estimate the variation explained due to different blood cell types [61] and no significant differences were found for the blood cell types for HRO-related analysis (Table S3) and, in the case of age-related analysis, we have included the estimated T cell (CD8+), T cell (CD4+), and B cell levels as covariates. The expression analyses were performed only in one of the two Spanish populations. In addition, the association analysis between age and DNA methylation has been focused on middle-aged subjects. Another limitation of this work would be the lack of children, youth and elderly subjects to replicate the age-related associations. However, this research is an initial study that enables the extrapolation of these findings to other populations. Moreover, the larger strength of the present work is that the results (associations between DNA methylation and age or the measures of HRO) are independent of gender; the selected CpG sites identified in the age and HRO-related analyses were significant in both cohorts and in the pooled population, and after controlling for several covariates besides gender. Moreover, the experimental design of the current work includes a dual approach in the same cells (the combination of DNA methylation and gene expression microarrays) for the search of HRO biomarkers. It also uses a type of biological sample (WBC) very easy to obtain and non-invasive.

## 3. Experimental Section

### 3.1. Subjects and Study Protocol

The current analysis was conducted within a subsample of 48 obese adults (48 ± 10 years old; BMI 36.2 ± 3.8 kg/m^2^; 46.8% female) that participated in the RESMENA (Metabolic Syndrome Reduction in Navarra) project which is a randomized controlled trial [62], and 25 subjects from the OBEPALIP study [63], which consisted on healthy women with an age range between 21 and 45 years old and a BMI between 27.5 and 36.40 kg/m^2^. The metabolic syndrome was diagnosed following the ATP III criteria [64]. Both studies were approved by the Ethics Committee of the University of Navarra. Consequently, all participants provided written, informed consent for participation in agreement with the Declaration of Helsinki (as revised in Hong Kong in 1989, in Edinburgh in 2000 and in Korea in 2008). DNA and RNA were obtained through a clinical study approved by the Ethics Committee of University of Navarra (project identification code: 48/2009 on 27 November 2009 (RESMENA) and 007/2009 on 26 February 2009 (OBEPALIP)) and appropriately registered at www.clinicaltrials.gov; NCT01087086 and NCT01138774.

### 3.2. Procedures

Anthropometric measurements (body weight, height and waist circumference) were conducted according to validated protocols, as previously described [62,63]. In order to evaluate HRO, the pooled population was assigned to two groups according to BMI classification and risk of obesity-related health problems [65]: “Low HRO” (Overweight (BMI: 25.0–29.9 kg/m^2^) and class 1 obesity (BMI: 30.0–34.9 kg/m^2^)) and “High HRO” (class 2 obesity (BMI: 35.0–39.9 kg/m^2^) and class 3 obesity (BMI > 40 kg/m^2^)).

Venous blood samples were drawn by venipuncture after a 12-h overnight fast. The EDTA—Plasma samples and WBC were separated from whole blood by centrifugation at 3500 rpm, 5 °C, 15 min (Model 5804R, Eppendorf, Humburg, Germany), and were frozen immediately at −80 °C until assay (WBC in buffy-coat with and without TRIzol reagent).

### 3.3. DNA Isolation and DNA Methylation Analysis

Genomic DNA was isolated from WBC by using the MasterPureTM DNA Purification Kit (Epicentre Biotechnologies, Madison, WI, USA) according to the manufacturer’s instructions. DNA was quantified using the PicoGreen^®^ dsDNA Quantitation reagent (Invitrogen, Carlsbad, CA, USA). Bisulfite modification of 500 ng genomic DNA was carried out using the EZ DNA methylation kit (Zymo Research, Orange, CA, USA) according to the manufacturer’s protocol.

Bisulfite-treated genomic DNA was whole-genome amplified, hybridized to Infinium Human Methylation 450K BeadChips (Illumina, San Diego, CA, USA) and scanned using the Illumina iScanSQ platform. The intensity of the images was extracted with the GenomeStudio Methylation Software Module (v 1.9.0, Illumina, San Diego, CA, USA). β-Values were computed using the formula β-Value = M/(U + M) where M and U are the raw “methylated” and “unmethylated” signals, respectively. β-Values were corrected for type I and type II bias using the peak-based correction. The data were normalized in R using a categorical Subset Quantile Normalization method (SQN) and probes associated with X and Y chromosomes were filtered out using the pipeline developed by Touleimat and Tost [66]. Probes containing Single Nucleotide Polymorphisms (SNPs) with a minor allele frequency (MAF) <0.001 in Iberian population and probes hybridizing multiple genomic locations (19,835) [67] were removed from the analysis. A total of 358,639 CpG sites were used in order to identify CpG sites associated with age and HRO. Moreover, we performed an additional analysis to estimate the variation explained due to different cell types [61] and no significant differences were found for blood cell types for HRO-related analysis (Table S3). However, the T cell (CD8+), T cell (CD4+) and B cell coefficients for age were significant, so these estimated cell types were included in the adjustment for age (Table S3). The limma package [68] for the R statistical software was used to compute a linear regression (for age outcome) or moderated F-statistic (for HRO) adjusted by the effect of confounding factors, such as gender, smoking, metabolic syndrome, the research group that made each study, and batch effect and T cell (CD8+), T cell (CD4+) and B cells for age-related analysis. Also, the age-cohort and HRO-cohort interaction were taken into consideration. Raw p values were adjusted using the Benjamini-Hochberg procedure [69] and an False Discovery Rate (FDR) cut-off of 0.05 for the age-related analyses and *p*-value cut-off of 0.01 in combination with methylation differences of 10% in the HRO-related analyses were used as statistical significant threshold.

### 3.4. Gene Ontology (GO) Analysis

Gene Ontology (GO) was used to investigate the biological relevance of the CpG sites that were associated with age or HRO [70]. GO annotations of genes were obtained from the Locus Link database of NCBI (http://www.ncbi.nlm.nih.gov/).

### 3.5. Gene Expression Analysis

A total of 24 RNAs were purified from WBC of the RESMENA study by using the TRIzol RNA isolation protocol (Life technologies, Foster City, CA, USA) and the integrity of isolated RNA was evaluated with the Experion chip electrophoresis unit (Bio-Rad Laboratories, Munich, Germany) following the manufacturer’s instructions. In all samples we evaluated the RNA quality indicator number (RQI), which was considered optimal when ranging from 7.9 to 10. A total of 500 ng of starting material was used as input for the Illumina TotalPrep Amplification Kit protocol (Life Technologies, Foster City, CA, USA).

Array-based gene expression analysis was performed with the Whole-Genome Assay technology (Illumina, San Diego, CA, USA). Labeled sample cRNA was hybridized to HumanHT-12 v4 Expression BeadChip Kit (Illumina, San Diego, CA, USA) and scanned using the Illumina HiScan™ SQ platform. The intensity of the images was extracted with the GenomeStudio Gene Expression Software Module (v1.9.0, Illumina, San Diego, CA, USA).

### 3.6. Statistical Methods

Data are expressed as means (standard deviations, SD), except as otherwise indicated. The characteristics of participants according to the two studies and HRO were compared. Differences in continuous values in Low *versus* High HRO subjects were performed by the Student’s *t*-test. Categorical variables were analyzed by χ^2^ test. Spearman correlations were fitted to evaluate the potential correlations of DNA methylation with age and expression levels. In addition, multiple linear regression models were performed to analyze the prediction of BMI (outcome) for selected CpG sites from HRO-related analysis adjusted by age, gender, smoking, metabolic syndrome, the research group that made each study, and batch effect. For stringency, only a *p*-value <0.05 was considered for analyses. Statistical analyses were performed using SPSS Statistics 19 software package (SPSS Inc., IBM, Somers, NY, USA).

## 4. Conclusions

As a conclusion, we have been able to replicate several findings from previous studies in two different Spanish cohorts, supporting an important role of DNA methylation in the age-related regulation of gene expression. We have described a significant influence of age on DNA methylation and mRNA levels of different genes in WBC (*i.e*., *ELOV2*, *HOXC4* and *PI4KB*) and found, on a trend level, novel associations between DNA methylation and measures of HRO in genes like *GPR133* and *ITGB5*. These data reinforce the idea that epigenetic variation has an important impact on aging and HRO. These results are a first step in understanding the modifications of the epigenome (and gene expression) in relation to age- and BMI-related health outcomes. However, further studies on the relevance of these and other CpG sites in relation to age and HRO should be performed.

## Supplementary Materials

Supplementary materials can be found at http://www.mdpi.com/1422-0067/16/08/16816/s1.
