# Supplementary material for: Differential DNA Methylation in Relation to Age and Health Risks of Obesity
Source: Int J Mol Sci. 2015 Jul 24;16(8):16816–32. doi: 10.3390/ijms160816816 (PMC4581172; doi:10.3390/ijms160816816)
Supplement: Supplementary file 1 [file ijms-16-16816-s001.zip › ijms-85569-Supplementary Information/ijms-85569-Table S3.pdf]

Supplementary Information

Table S3. Estimated Regression Coefficients for age and HRO in pooled population.

Age	Est	SE0	SE1	SE2	Zscore	*p*-Values
(Intercept, γ0)	−0.01231	0.02858	0.02823	0.02886	−0.4263	0.669865
T Cell (CD8+)	0.43564	0.13114	0.14679	0.14703	2.963	0.003047
T Cell (CD4+)	−0.42751	0.13095	0.1217	0.13988	−3.0562	0.002242
NK Cell	−0.07706	0.03764	0.0416	0.04161	−1.8518	0.064055
B Cell	−0.07617	0.02172	0.03224	0.0324	−2.3511	0.018719
Monocyte	−0.03749	0.03753	0.04363	0.04556	−0.8228	0.410599
Granulocyte	0.23294	0.0373	0.11958	0.11932	1.9521	0.050921
**HRO**	**Est**	**SE0**	**SE1**	**SE2**	**Zscore**	***p*-Values**
(Intercept, γ0)	−0.2735	0.8333	0.6222	0.6208	−0.4406	0.6595
T Cell (CD8+)	0.1167	3.8234	3.3733	3.086	0.0378	0.9698
T Cell (CD4+)	−2.120	3.8181	2.568	2.9295	−0.7236	0.4693
NK Cell	−0.3187	1.0975	0.9321	0.937	−0.3401	0.7338
B Cell	0.1743	0.6333	0.6583	0.6704	0.2600	0.7948
Monocyte	−0.6448	1.0943	0.9498	0.9738	−0.6622	0.5079
Granulocyte	2.6731	1.0874	2.5669	2.5735	1.0387	0.2989

Est = Regression coefficient estimate (×100%); SE0 = Naive standard error (×100%); SE1 = Single-bootstrap standard error (×100%); SE2 = Double-bootstrap standard error (×100%); Zscore = Statistical measurement of a score’s relationship to the mean in a group of scores (×100%); NK = Natural killer; *p*-values were computed using SE2.
